# Biodegradable Hydrogels Loaded with Magnetically Responsive Microspheres as 2D and 3D Scaffolds

**DOI:** 10.3390/nano10122421

**Published:** 2020-12-03

**Authors:** Estela O. Carvalho, Clarisse Ribeiro, Daniela M. Correia, Gabriela Botelho, Senentxu Lanceros-Mendez

**Affiliations:** 1 Centre of Physics, University of Minho, 4710-057 Braga, Portugal; eocarvalho@fisica.uminho.pt (E.O.C.); d.correia@fisica.uminho.pt (D.M.C.); 2 Centre of Biological Engineering, University of Minho, 4710-057 Braga, Portugal; 3 Departamento de Química e CQ-VR, Universidade de Trás-os-Montes e Alto Douro, 5001-801 Vila Real, Portugal; 4 Centro de Química, Universidade do Minho, 4710-057 Braga, Portugal; gbotelho@quimica.uminho.pt; 5 BCMaterials, Basque Center for Materials, Applications and Nanostructures, UPV/EHU Science Park, 48940 Leioa, Spain; lanceros@fisica.uminho.pt; 6 IKERBASQUE, Basque Foundation for Science, 48009 Bilbao, Spain

**Keywords:** hydrogel, magnetoelectric spheres, tissue engineering, bone, mechano-electric stimuli

## Abstract

Scaffolds play an essential role in the success of tissue engineering approaches. Their intrinsic properties are known to influence cellular processes such as adhesion, proliferation and differentiation. Hydrogel-based matrices are attractive scaffolds due to their high-water content resembling the native extracellular matrix. In addition, polymer-based magnetoelectric materials have demonstrated suitable bioactivity, allowing to provide magnetically and mechanically activated biophysical electrical stimuli capable of improving cellular processes. The present work reports on a responsive scaffold based on poly (L-lactic acid) (PLLA) microspheres and magnetic microsphere nanocomposites composed of PLLA and magnetostrictive cobalt ferrites (CoFe_2_O_4_), combined with a hydrogel matrix, which mimics the tissue’s hydrated environment and acts as a support matrix. For cell proliferation evaluation, two different cell culture conditions (2D and 3D matrices) and two different strategies, static and dynamic culture, were applied in order to evaluate the influence of extracellular matrix-like confinement and the magnetoelectric/magneto-mechanical effect on cellular behavior. MC3T3-E1 proliferation rate is increased under dynamic conditions, indicating the potential use of hydrogel matrices with remotely stimulated magnetostrictive biomaterials for bone tissue engineering.

## 1. Introduction

The appearance of bone disorders and diseases is incessantly increasing due to population aging. The development of a method to properly repair damaged tissue remains a medical challenge [1]. Tissue engineering (TE) pursues to overcome the limitations of conventional medical therapies by applying biochemical, mechanical and electromechanical cues in order to recreate the tissue microenvironment improving tissue regeneration [2,3,4]. TE combines engineered biomaterials, called scaffolds, supplemented with cell and/or biological entities, such as growth factors and hormones. These endogenous entities are typically described as critical stimuli; however, the difficulty in controlling dose administration and off-target delivery restrict their clinical potential [5]. Currently, many efforts are focused on improving scaffolds characteristics as it has been demonstrated that scaffolds with conventional properties, such as easy integration with the surrounding tissue, good nutrients/metabolites diffusion and an adequate filling of the injured site, are not efficient enough for proper tissue regeneration. In fact, engineered tissue success depends critically on the scaffold’s ability to mimic native features of the damaged tissue [6,7].

Hydrogel-based matrices are attractive scaffolds to tissue-regenerative processes [8]. Cell-scaffold interaction is modulated by the hydrogel due to their high-water content, similar to the native extracellular matrix. The ability to fill any space and the fact that invasive surgery is not necessary for implantation are also advantages [9]. Moreover, these kinds of cell support can be loaded with specific active molecules/materials to add tissue-specific cues [10,11,12]. Further, the hydrogel systems are easily tuned in terms of porosity and stiffness, which have been proven to affect stem cell fate [13]. In fact, mechanical cues provided by hydrogel planar matrices regulate cellular expansion [14] and osteogenic and adipogenic differentiation [15] of mesenchymal stem cells. Much of these works have been performed in two-dimensions (2D) when cells are seeded over the hydrogel structure. However, cells naturally occupy a complex three-dimensional (3D) network, which can result in different cellular behaviors compared to 2D cultures [16].

Regarding bone tissue features, it is a dynamic tissue that adapts itself to mechanical, environmental cues. The mechanotransduction process has been identified as one of the main responsible for bone adaption [17]. Thus, mechanical forces are capable of triggering the activation of intracellular signaling cascades involved in bone repair and regeneration processes [7,18,19]. Addressing these needs, magnetostrictive materials are considered an efficient biomimetic approach. These materials undergo deformation as a response to an applied magnetic field, allowing to dynamically trigger mechanotransduction processes within the cells [20]. Further, when embedded in piezoelectric materials, this magneton transduction will further trigger a surface potential variation of the piezoelectric material, which has been proven particularly suitable for bone [21] and muscle [22] tissue engineering. A previous study combined CoFe_2_O_4_/poly(vinylidene fluoride) (PVDF) with a methacrylated gellan gum hydrogel and demonstrated the suitability of the incorporation of magnetoelectric spheres into the hydrogel, obtaining a magnetically responsive scaffold [11]. However, the influence of the mechano-electrical stimuli was not evaluated, neither the use of biocompatible nor biodegradable polymers, such as poly(l-lactide) (PLLA), which also present a piezoelectric response that can be activated magnetically with magnetostrictive nanofillers through the magnetoelectric effect [23].

Hence, in this work, the synergic combination of a hydrogel scaffold with biophysical cues, provided by magnetoelectric microspheres, in osteoblasts proliferation was evaluated. For that, in both 2D and 3D cell cultures, two different cell culture conditions were created. In the first condition, cells grew in a typical 2D monolayer over the hydrogel, while in 3D culture, the cells were embedded in the hydrogel matrix as a bone-mimicking structure [18]. The activation of the magnetostrictive content of the scaffolds was carried out with a magnetic bioreactor. Thus, the scaffold will ultimately support tissue recovery by means of a combined stimulus.

## 2. Materials and Methods

### 2.1. Materials

PLLA (217.000–225.000 g·mol^−1^) and poly(vinyl alcohol)—PVA—(13.000–23.000 g·mol^−1^, 98% hydrolyzed) were purchased from Purac and Sigma Aldrich, respectively. CoFe_2_O_4_ with dimensions ranging from 35 to 55 nm were acquired by Nanoamor (Katy, TX, USA). For the PLLA dissolution, chloroform (98% purity) from Merck (Sintra, Portugal) was used. The hydrogel based on methacrylated gellan gum was supplied from Stemmaters (Guimarães, Portugal).

### 2.2. Preparation and Characterization of PLLA Microspheres

Neat and magnetic PLLA microspheres were produced by the oil–water emulsion technique as described in [24]. Briefly, PLLA was dissolved in chloroform in a concentration of 3% (*w*/*v*) and then mixed with a surfactant solution of 0.5% (*w*/*v*) of PVA. The PLLA/PVA ratio was selected based on previous works [24,25]. The suspension was magnetically stirred overnight at room temperature (RT) to ensure solvent evaporation. The obtained microspheres were washed with ultrapure water for 3 h. Both PLLA microspheres, neat microspheres and PLLA microspheres with magnetic content (magnetic microspheres) were prepared by the aforementioned procedure. For magnetic microspheres, CoFe_2_O_4_ nanoparticles were incorporated immediately after PLLA dissolution.

### 2.3. Characterization of the PLLA Microspheres

The produced microspheres were characterized in terms of size and morphology, physicochemical and thermal properties, as well as with respect to the magnetic properties [24].

The size distribution and morphology of the microspheres were evaluated by scanning electron microscope (SEM, Quanta 650, from FEI equipment, Hillsboro, Oregon, USA) at 1 kV. Samples were added to aluminum pin stubs with conductive carbon adhesive tape (PELCO Tabs™, Agar scientific, Essex, United Kingdom) and sputter-coated with gold (Polaron, model SC502). ProSuite software was used to acquire the results. The average diameter was calculated over approximately 50 microspheres using the SEM images in ImageJ software (bundled with 64-bit Java 1.8.0_172).

The hydrodynamic size was analyzed using dynamic light scattering (DLS, Zetasizer NANO ZS-ZEN3600 equipment, Malvern). Before DLS measurements, samples were centrifuged at 2500 rcf for 5 min to homogenize them. Then, with appropriated dilutions in ultrapure water to avoid multi scattering events, six measurements were performed at 25 °C for each sample.

Fourier-transform infrared analysis (FTIR, Jasco 4100 equipment, Easton, Maryland, USA) coupled with an attenuated total reflectance (ATR) accessory were carried out. The spectra were acquired at room temperature from 4000 to 400 cm^−1^ and collected after 64 scans with a resolution of 4 cm^−1^.

Thermal characterization was accessed by differential scanning calorimetry (DSC, Mettler Toledo 823 instrument, Columbus, OH, USA). The samples were placed into aluminum pans and heated at a heating rate of 10 °C·min^−1^ under a nitrogen purge.

The magnetic response was assessed using a vibrating sample magnetometer (VSM, MicroSense EZ7 equipment, Lowell, MA, USA) from −6000 to 6000 Oe to measure the room temperature magnetic hysteresis loops up to magnetization saturation. The CoFe_2_O_4_ filler content within the microspheres was determined through Equation (1), based on the saturation magnetization of the microspheres (*saturation magnetization*) and the saturation magnetization of CoFe_2_O_4_ powdered particles (*saturation magnetization pristine*):

(1) $${\mathrm{CoFe}}_{2}O_{4}\;\mathrm{wt}\;{\%}_{\mathrm{microspheres}}=\frac{Saturation\;magnetization}{Saturation\;magnetization\;pristine}\times 100$$

### 2.4. Cell Culture

Two main tests were performed to assess cytotoxicity and indirect cell proliferation. First, cytotoxicity was considered for both microsphere types, and then the indirect proliferation of pre-osteoblasts cultured above and within the hydrogel matrix, containing neat or magnetic microspheres, in static and dynamic conditions was evaluated. The hydrogel matrix was obtained by diluting it in distilled water at a concentration of 1.25 wt %, as recommended by commercial instructions. Then, 10 mg·mL^−1^ of neat or magnetic microspheres powder were mixed vigorously for 10 s to homogenize the solution.

Before any assay, neat and magnetic microspheres were sterilized. For that, dry microspheres were placed in Eppendorf tubes containing phosphate buffer saline (PBS) solution and, after they are dry, exposed to UV radiation for 30 min. The hydrogel did not need to be sterilized as it was supplied sterile.

#### 2.4.1. Cytotoxicity Assessment of PLLA Microspheres

The microspheres cytotoxicity evaluation was conducted with an adaptation of the ISO 10993-5 standard test method. For this, a previously sterilized portion of both microspheres (10 mg·mL^−1^) was immersed in a 24-well tissue culture polystyrene plate containing Dulbecco’s Modified Eagle’s medium (DMEM, Biochrom, Berlin, Germany) with 4.5 g·L^−1^ glucose, 10% fetal bovine serum (FBS, Biochrom, Berlin, Germany) and 1% penicillin/streptomycin (P/S, Biochrom), at 37 °C in 95% humidified air containing 5% CO_2_ and incubated for 24 h. Then, 20% (*v*/*v*) dimethyl sulfoxide (DMSO, Sigma Aldrich, Sintra, Portugal) and cell culture medium was used as the negative and positive control, respectively. Simultaneously, L929 fibroblast cells were plated in a 96-well tissue culture polystyrene plate with a density of 3 × 10^4^ cells·mL^−1^ (volume of 100 µL/well). Then, cells were incubated for 24 h to ensure the attachment on the plate. After 24 h, the culture medium was removed, and the medium that was in contact with microspheres was added to each well (100 µL). Then, cells were incubated for 72 h, and the indirect cell viability was evaluated using a 3-(4,5-dimethylthiazol-2-yl)-2,5-diphenyltetrazolium bromide (MTT) assay. For that, after this time point, the medium was removed and added a new medium containing 10% MTT solution. After 2 h of incubation, formed MTT crystals were dissolved with DMSO, and the optical density was measured at 570 nm with a microplate reader (Biotech Synergy HT, Winooski, VT, USA). The MTT assay quantifies viable cells as they convert MTT into purple-colored formazan crystal, which is dissolved by DMSO.

#### 2.4.2. Cell Proliferation Assays at Static and Dynamic Conditions

**Figure 1:**
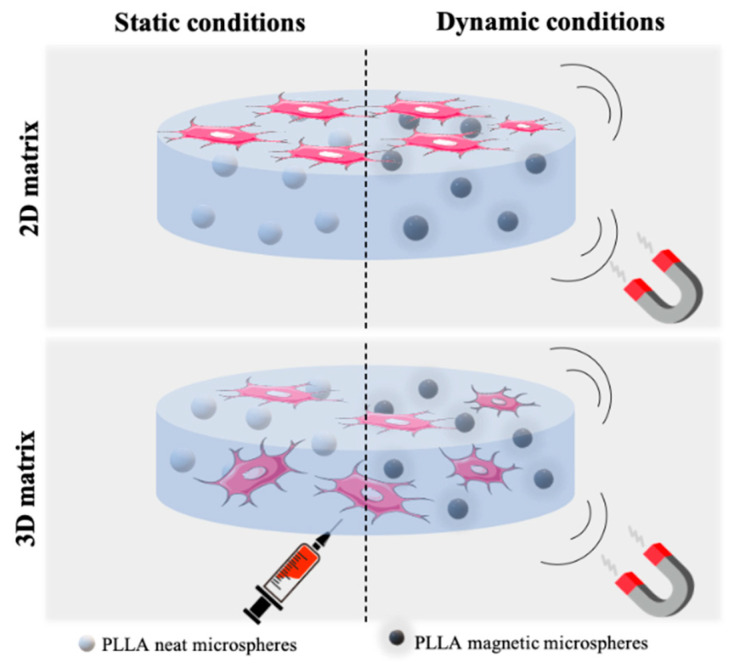
Schematic representation of cell culture conditions.

Cell culture assays were performed with MC3T3-E1 preosteoblast cell (Riken bank, Tsukuba, Japan). First of all, MC3T3-E1 was grown in a 75 cm^2^ cell-culture flask with DMEM medium, containing 1 g·L^−1^ glucose, 10% FBS and 1% P/S. The flask was placed in a 37 °C incubator under 95% humidified air and 5% CO_2_ conditions, and, after two days, the medium was changed until reaching 60–70% confluence to be trypsinized (0.05% trypsin-EDTA, Biochrom, Berlin, Germany). For 2D matrix cell culture, a cell suspension with a density of 1.6 × 10^6^ cells·mL^−1^ was seeded on the hydrogel surface (approximately 2 × 10^4^ cells/cm^2^), which was previously mixed with neat or magnetic microspheres. A drop method was applied to avoid the cells seeding on the plate rather than on the hydrogel. The same procedure occurred for 3D matrix cell culture; however, in this case, the cell suspension was carefully injected into the hydrogel matrix (approximately 2 × 10^4^ cells in 50 µL of hydrogel) (Figure 1). The well volume was completed with medium and incubated.

After 24 h, cell adhesion was evaluated. For that, two replicates of each condition were washed with PBS 1×, then fixed with 4% formaldehyde (Panreac) and subjected to immunofluorescence staining. For that, 1 µg·mL^−1^ of phalloidin tetramethyl rhodamine (TRITC, Sigma Aldrich, Sintra, Portugal) solution was used to stain the cell’s cytoskeleton (45 min at RT) and 1 μg·mL^−1^ of a 4,6-diamidino-2-phenylindole (DAPI, Sigma Aldrich, Sintra, Portugal) solution to stain the cell’s nucleus (5 min at RT). Samples were washed with PBS 1× between each stain process and at the end.

Still, at this time point, three other replicates of each condition were assayed to assess indirect cell proliferation. Thus, samples were subject to 3-(4,5-dimethylthiazol-2-yl)-5-(3-carboxymethoxyphenyl)-2-(4-sulfophenyl)-2H-tetrazolium (MTS, Promega, Madison, WI, USA) assay. Similar to MTT, the MTS test is a coloring method that allows the determination of cell viability based on NADPH or NADP-assisted bioreduction in living cells. Briefly, the samples were placed in a new plate and further incubated with MTS solution in a 1:5 ratio at 37 °C for 2 h. Then, 100 µL of each well was transferred to a 96-well plate and measured the optical density at 490 nm using a spectrophotometric plate reader (Biotech Synergy HT, Winooski, VT, USA). A plate with 2D and 3D cell cultures containing neat microspheres was maintained in static conditions (cell culture without any applied stimuli), and another one, with both culture containing magnetic microspheres, was transferred onto a home-made bioreactor system, which provides dynamic conditions (cell culture under magnetic stimulation), for up to 48 h. The magnetic stimulation cycle was applied based on previous studies (1 Hz frequency and 1 mm amplitude for an active time of 16 h under stimulation followed by a non-active time of 8 h) [18,20]. Lastly, after 48 h, cell viability was again assessed through MTS assay, as described above, in samples of static and dynamic conditions.

### 2.5. Data Analysis

The obtained results are presented as the average of individual measurements with the respective standard deviations. Graph Pad Prism Version 7 for Windows (Graph Pad Software, San Diego, CA, USA) was used to analyze the results and the statistical significance, using two-way ANOVA followed by Tukey’s test. Differences were considered to be statistically significant when *p*-value < 0.05.

## 3. Results and Discussion

### 3.1. Morphological and Physical-Chemical Characterization of PLLA Microspheres

**Figure 2:**
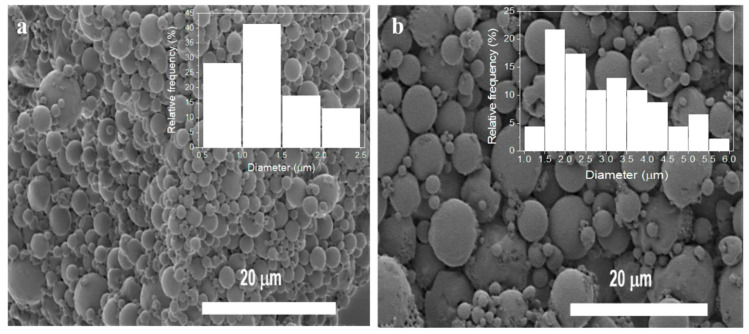
Morphology of (**a**) neat and (**b**) magnetic microspheres and corresponding size distribution obtained by SEM and ImageJ software, respectively.

The morphology of the PLLA microspheres, produced by an oil–water emulsion to integrate within the responsive hydrogel scaffold, was analyzed by SEM. Figure 2 shows representative SEM images, as well as the corresponding size distribution. A smooth and homogenous spherical surface, without cavities or distortions, is observed in both images (Figure 2a,b), demonstrating that the polymer solution was well dispersed in the aqueous phase, without solids precipitation. The screening of neat PLLA microspheres (Figure 2a) shows a size distribution ranging between 0.7 and 2.35 μm with an average size of 1.3 ± 0.4 μm. On the other hand, magnetic microspheres (Figure 2b) exhibited a slightly wider size distribution, ranging between 1.4 and 5.6 μm, and, consequently, a larger average size, 3 ± 1 μm, also suggesting a more heterogeneous size distribution. The presence of a magnetic core composed of CoFe_2_O_4_ nanoparticles agglomerated inside the magnetic microspheres deflects the average diameter to higher values.

**Figure 3:**
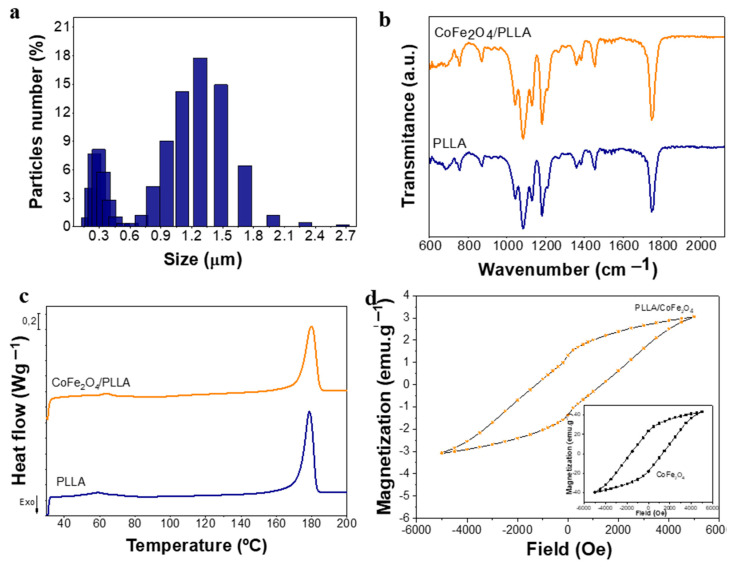
Microspheres characterization: (**a**) hydrodynamic size distribution of neat microspheres obtained by dynamic light scattering (DLS) measurements; (**b**) FTIR-ATR spectra and (**c**) differential scanning calorimetry (DSC) curves of both synthesized microspheres; and (**d**) magnetization curves of magnetic microspheres and CoFe_2_O_4_ pure powder (inset) obtained by vibrating sample magnetometer (VSM) experiments.

With respect to the hydrodynamic size and distribution, Figure 3a shows the distribution of the neat microspheres obtained by DLS measurements in ultrapure water and neutral pH. The hydrodynamic average size corresponds to 1.39 ± 0.02 μm, a similar value to the one obtained by SEM images (1.3 ± 0.4 μm), reflecting the hydrodynamic stability at a neutral pH as the microspheres were centrifuged for DLS measurements. A decrease in standard deviations can result from this. Apparently, neat PLLA microspheres show a different population distribution. This fact is ascribed to the appearance of moderated heterogeneity, according to a polydispersion index of 0.21 ± 0.03. Concerning magnetic microspheres, they possess a very high sedimentation rate preventing the assessment of their hydrodynamic stability. This fact has already been reported previously, being concluded that the particles agglomerate and deposit during measurements [24].

The physicochemical and thermal properties of the produced spheres were also evaluated. Figure 3b shows the spectra of neat and magnetic microspheres, both of which present the same absorption bands. The absorption bands at 750 cm^−1^ and 870 cm^−1^ are characteristics of the crystalline and amorphous PLLA phase, respectively [26]. There is also an absorption band at 921 cm^−1^ indicating the coupling of C‒C backbone stretching with CH_3_ rocking mode and specifying the presence of α-crystals [27]. The presence of an absorption band at 1085 cm^−1^, as well as bands close to 1450 cm^−1^, correspond to the stretching vibration of the methyl group (C‒H) [28]. Then, bands at the 1184 cm^−1^ region are characteristic of asymmetric C‒O‒C asymmetric stretching linked with CH_3_ rotation [29]. Finally, regions with the highest frequency illustrate two bands of large relevance at 1750 and 2999 cm^−1^, corresponding to the stretching vibration of ester carbonyl (C=O) and stretching vibration of the CH_3_ group, respectively [30]. Absorption bands are coincident with those mentioned in the literature, suggesting the successful processing of the PLLA microspheres and showing that the chemical properties of PLLA also does not change after the inclusion of the nanoparticles, as well as with the use of PVA as a surfactant (no PVA typical bands were observed in the FTIR spectra).

The thermal behavior of neat and magnetic microspheres was analyzed after DSC measurements. Both thermograms are presented in Figure 3c. Neat microspheres thermogram exhibits endothermic peaks at ≈60 °C, corresponding to the glass transition, and at ≈179 °C, which represents the melting transition. Regarding magnetic microspheres, a shift towards higher temperatures was observed, which demonstrates the CoFe_2_O_4_ filler nucleation effect during PLLA crystallization [24]. In these microspheres, glass transition assumes 65 °C while melting transition reached a maximum at 181 °C, in the same line of the data obtained for PLLA and magnetic composites [24].

Regarding magnetic properties, CoFe_2_O_4_/PLLA microspheres were evaluated at room temperature by VSM. Figure 3d shows the magnetization curves of magnetic microspheres and CoFe_2_O_4_ nanoparticles. The magnetization curves increase with the increasing magnetic field until it reaches saturation. For magnetic microspheres, the maximum saturation magnetization is reached at 3.07 emu·g^−1^. Comparing the saturation magnetization values of both hysteresis loops through equation 1, the amount of CoFe_2_O_4_ nanoparticles dispersed in the polymer matrix is estimated to be 6.40% instead of the 10% included in the solution. The encapsulation efficiency of ≈64% is due to the higher density of the CoFe_2_O_4_ nanoparticles in comparison with the PLLA polymer, leading to faster sedimentation of the particles in the solution during the microsphere formation.

### 3.2. Cytotoxicity Assessment of PLLA Microspheres

**Figure 4:**
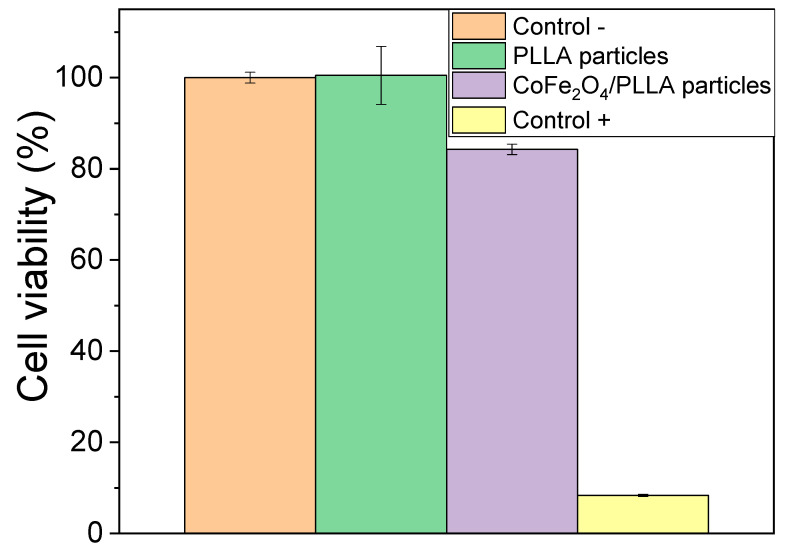
Cytotoxicity assay results of the 3T3 fibroblast cells in contact with the as-prepared extraction media exposed to the poly (L-lactic acid) (PLLA) and CoFe_2_O_4_/PLLA particles after 72 h (relative metabolic activity was presented as the percentage of the negative control with n = 4 ± standard deviation).

Since these magnetic nanoparticles can be toxic [31,32], a cytotoxicity assay was performed in order to evaluate the effectiveness of magnetic nanoparticles encapsulation in the PLLA polymer. As previously described, microspheres were placed in contact with the cell culture medium for 24 h and then this medium was used in the cell culture of 3T3 fibroblasts. After 72 h, the different wells were subjected to MTT experiments, and the results are shown in Figure 4.

Regarding the obtained results, it is verified that the PLLA spheres are no cytotoxic and that the magnetic nanoparticles were well encapsulated once the metabolic activity is higher than 70%. The small decrease of the value of metabolic activity for the CoFe_2_O_4_/PLLA spheres can be due to the presence of some magnetic particles at the surface of the spheres, but without affecting their viability.

### 3.3. Cell Proliferation Assays at Static and Dynamic Conditions

**Figure 5:**
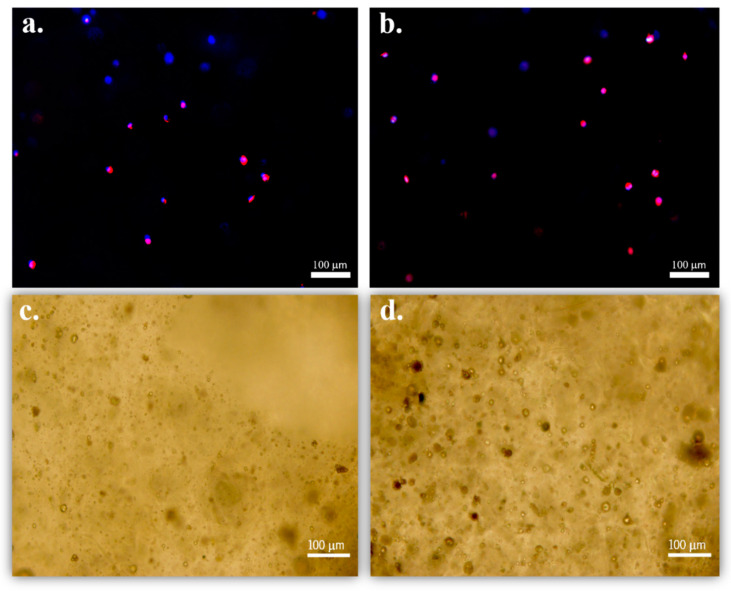
MC3T3-E1 adhesion to the hydrogel containing (**a**) neat and (**b**) magnetic microspheres after 24 h, under static conditions and in a 3D matrix cell culture; and (**c**) PLLA spheres and (**d**) CoFe_2_O_4_/PLLA spheres uniformly dispersed in the hydrogel.

Preosteoblast adhesion to hydrogel composites in a 3D matrix cell culture was evaluated using fluorescence microscopy after 24 h under static conditions. It is to notice that the immunofluorescence images (Figure 5) show that the cells present a spherical shape, suggesting that after 24 h, cell elongation has not yet occurred. Analogously to 2D hydrogel culture or in flat scaffolds, the cells in 3D hydrogel matrices are forced to acquire a spheroidal shape due to a 3D-enhancing mechanism of hydrogel encapsulation. Regardless of the cell type or native morphology, cells take a long time to elongate [33,34]. Regarding the 2D matrix, it is possible to verify that the spreading of the cells is faster than in the 3D matrix.

**Figure 6:**
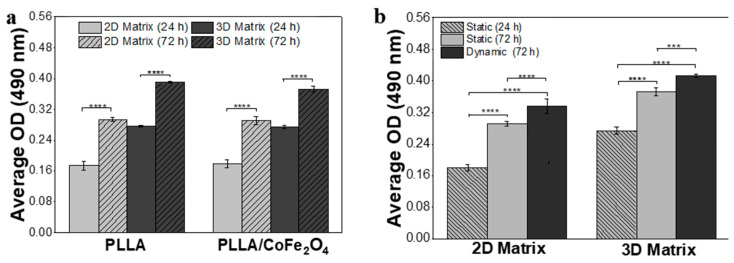
MC3T3-E1 proliferation in the hydrogel matrix containing (**a**) neat and magnetic microspheres, under the static condition and in both matrices; and (**b**) magnetic microspheres under dynamic conditions and in both matrices. *** *p* < 0.001; **** *p* < 0.0001.

After ensuring cell adhesion, the indirect proliferation rate was assessed, and then we selected the preferred conditions for cell growth. Thus, samples of 2D and 3D cell culture, under static and/or dynamic conditions, were submitted to an MTS assay after 24 and 72 h timepoints. Figure 6 shows the corresponding average optical densities (OD).

Through Figure 6a, cell proliferation is achieved in any condition (type of microsphere and cell culture), highlighting that this graph only reports cell culture under static conditions. All averages OD corresponding to 72 h is significantly higher than those obtained at 24 h timepoint. Further, it is verified, since the same number of cells are seeded in both matrices, that a higher number of cells adhered in the 3D matrix. Observing the OD in 2D and 3D matrices, it is to notice that this is significantly superior when cells grow surrounded by a hydrogel matrix in both time points. This is expected as in a 3D matrix, and cells are seeded in several layers. However, comparing the proliferation rate of both conditions after 72 h, cell culture increases more ≈27% in a 2D matrix than in a 3D matrix. This phenomenon occurs in neat and magnetic microspheres, suggesting that, in the absence of stimulation, cell proliferation is independent of microspheres’ nature. Moreover, these results corroborate the previous ones since no physical or chemical difference was registered in the characterization of neat and magnetic microspheres.

The evaluation of preosteoblast proliferation under external stimulation, provided by a magnetic bioreactor (dynamic parameters mentioned above), is shown in Figure 6b. This experiment was carried out on responsive scaffolds formed by 2D or 3D matrices containing magnetic microspheres. The results again showed that the OD average for the 3D matrix is higher than that obtained in the 2D matrix. Analogously to Figure 6a, cell proliferation rate, in dynamic conditions after 72 h, is ≈35% higher when MC3TC-E1 grows on a planar composite matrix. Finally, for both cell culture matrix, comparing the OD results of static and dynamic conditions at the same 72 h timepoint, it is shown that cells are positively influenced by magnetic stimulation.

Magnetic stimulation leads to two effects: magnetostrictive variations of the magnetic nanoparticles that are transmitted through the PLLA polymer matrix and lead to surface charge variations through the magnetoelectric effect [23], and the slight vibration of the PLLA microspheres within the hydrogel due to magnetic force. Thus, dynamic cell culture condition allows mimicking the mechanical stress variations detected by osteoblasts throughout the day due to natural body movements. In turn, the results are in line with those reported by literature for piezoelectric dynamic stimulation, where mechanical variations further lead to surface change variations of the scaffolds [18,35].

In this way, the combination of magnetoelectric spheres with hydrogels can improve cell regeneration through mechano-electrical stimulation, demonstrating that these systems can successfully mimic the complex natural electromechanical microenvironments found in the human body. Finally, it is no notice CoFe_2_O_4_ particles were selected as magnetic responsive material due to its superior magnetic characteristics when compared with related ferrites. As PLLA degradation is slow (>1 year), the developed system can be used for low time cell cultures (e.g., in vitro studies) where the degradation of the polymer is not relevant. For eventual in vivo assays, where the degradation of the polymer is expected to play a relevant role, the magnetic particles can be replaced for nontoxic ones, such as magnetite Fe_3_O_4_.

## 4. Conclusions

Neat and magnetic microspheres based on PLLA polymer were prepared by oil–water emulsion processes. For magnetic microspheres, magnetostrictive CoFe_2_O_4_ nanoparticles were used. The spherical form of the produced spheres was confirmed by SEM images. Regarding physic-chemical and thermal properties, both microspheres show similar FTIR spectra and DSC characteristics among them. The magnetic characteristics of the microspheres were confirmed, and the encapsulation efficiency of 64% was obtained. Cytotoxicity assays showed the viability of both microspheres for the biomedical applications as well as their suitable incorporation in the hydrogel. The indirect proliferation assays demonstrate the ability of the hydrogel matrix as a scaffold, as well as the suitability of two promising approaches for cell culture—2D and 3D matrices. Moreover, it was proven the efficiency of magneto-mechanical actuation on the preosteoblasts proliferation under dynamic conditions.
